# Vortioxetine Improves Brain Glymphatic System Function, Functional Connectivity, and Cognitive Functions in Major Depressive Disorder

**DOI:** 10.1155/da/1990117

**Published:** 2025-08-30

**Authors:** Zixuan Guo, Xinyue Tang, Shuming Zhong, Guanmao Chen, Pan Chen, Chao Chen, Ruoyi Chen, Li Huang, Yanbin Jia, Ying Wang

**Affiliations:** ^1^Medical Imaging Center, First Affiliated Hospital of Jinan University, Guangzhou, China; ^2^Department of Medical Imaging, Third Affiliated Hospital of Southern Medical University, Guangzhou, China; ^3^Department of Radiology, The Affiliated Hospital of Southwest Medical University, Luzhou, China; ^4^Department of Psychiatry, First Affiliated Hospital of Jinan University, Guangzhou, China

**Keywords:** cognitive functions, glymphatic system, major depressive disorder, resting-state MRI, vortioxetine

## Abstract

**Background:** The therapeutic effects of vortioxetine on mood and cognition have been documented in major depressive disorder (MDD). This study aims to examine whether vortioxetine can improve brain glymphatic system function and connections among functional brain networks and to explore the underlying relationships among these changes.

**Methods:** A total of 34 patients with MDD and 41 healthy controls (HCs) were recruited in the study. All participants underwent mood and cognitive assessment, and diffusion tensor imaging (DTI) and resting-state functional MRI scans at baseline and 8-week follow-up. The DTI analysis along the perivascular space (DTI-ALPS) index, and functional connectivity (FC) were assessed. Cognitive assessment was conducted using the Chinese version of Measurement Consensus Cognitive Battery (MCCB). Correlation analysis was subsequently performed to explore underlying association among these indexes.

**Results:** Compared to HCs, patients with MDD showed decreased DTI-ALPS indexes at baseline; patients with MDD showed increased the default mode network (DMN) FC between the posterior cingulate cortex (PCC)–precuneus; patients with MDD displayed decreased attention/vigilance, verbal learning, visual learning, social cognition, and global cognition. Treatment with vortioxetine, patients with MDD displayed reduced depressive symptoms, increased DTI-ALPS indexes, decreased DMN FC, and improved attention/vigilance, verbal learning, visual learning, social cognition, and global cognition. Moreover, the increased DTI-ALPS indexes correlated with improved global cognition, and decreased DMN FC in MDD, respectively.

**Conclusions:** The current study indicated vortioxetine improves glymphatic system function and brain connections within the DMN in MDD. Furthermore, the restoration of glymphatic function is linked to improved brain function and cognition.

**Trial Registration**: ClinicalTrials.gov identifier: ChiCTR2200057820

## 1. Introduction

Major depressive disorder (MDD) is a leading disabling psychiatric disorder worldwide, marked by profound and persistent depressive symptoms and cognitive impairments [1]. Cognitive functions may worsen even after depressive symptoms have remitted [2]. As a result, cognitive deficit has been acknowledged as an important focus in depression therapy [2, 3]. Vortioxetine is an effective and well-tolerated antidepressant, functioning through the inhibition of the serotonin transporter and the modulation of various serotonin receptors [4, 5]. Accumulating evidence has suggested that vortioxetine could improve depressive symptoms and enhance cognitive performance in MDD [4, 5]. Notably, the cognitive benefits associated with vortioxetine appear to be largely a direct treatment effect rather than merely a consequence of improvements in depressive symptoms [5]. However, the underlying neuropathological mechanisms of these kinds of improvements remain to be elucidated.

The glymphatic system, characterized by the encasement of vascular conduits by astrocytes, functions as a critical mechanism for the removal of metabolic waste from the central nervous system, thereby highlighting its essential role in maintaining neurological health and function. Research in both preclinical and clinical settings has noted glymphatic dysfunction in neurological and psychiatric conditions like Alzheimer's disease [6], dementia [7], MDD [3], and schizophrenia [8]. The glymphatic system has been detected through multiple invasive methodologies, including ex vivo fluorescent microscopy and in vivo two-photon imaging in animals, alongside intrathecal contrast medium-enhanced MRI and dynamic positron emission tomography in humans. More recently, the diffusion tensor imaging along the perivascular space (DTI-ALPS) index and the perivascular space (PVS) volume have offered noninvasive and reliable methodologies for mapping the human glymphatic system, thereby facilitating the assessment of its functional competence in the context of disorder. Researchers found lower DTI-ALPS index in neurological disorders [9–11] and reduced DTI-ALPS index was correlated with cognitive impairments [12]. Additionally, neuroinflammation, characterized by microglia and astrocyte activation, has emerged as a critical pathophysiological mechanism underlying MDD [13, 14]. The release of inflammatory factors from activated microglia may influence glymphatic clearance, potentially modulating neuroinflammation in a feedback mechanism [15, 16]. A previous study has found decreased DTI-ALPS index in MDD relative to healthy controls (HCs) [17]. Nevertheless, the findings of the previous study were preliminary and cross-sectional in design [17]. To date, no research has been conducted to investigate the therapeutic effects of antidepressant on glymphatic function in patients with MDD.

The emergence of MRI technology has allowed researchers to identify subtle functional alterations in patients with MDD and has facilitated the elucidation of the relationship between brain abnormalities and the diverse clinical presentations of patients [18–20]. Accumulating evidence has implicated that four large-scale functional brain networks are crucial in the field of psychiatry, including the default mode network (DMN), salience networks (SNs), executive control network (ECN), and affective (limbic) network (AN) [21, 22]. MDD may not be limited to specific isolated brain regions; rather, it may originate from disruptions in resting-state functional connectivity (FC) among four large-scale functional brain networks [21, 22], with particular emphasis on the DMN [23, 24]. The DMN is associated with internally-oriented and self-referential thought [18, 19]. Disrupted internetwork connectivity in the DMN in patients with MDD may be associated with the prevalent negative self-referential thinking, rumination and difficulties in emotion regulation, and may contribute to the pathophysiological mechanisms of MDD [18, 19]. However, limited research has concentrated on the therapeutic effects on FC among brain networks in MDD. A large-scale study found that decreased FC among brain networks would associate with medication treatment effects in recurrent MDD patients [25]. A previous study indicated that pharmacological intervention with duloxetine or escitalopram significantly reduced FC among the DMN, ECN, and SN in medication remitters with MDD [26]. Nevertheless, whether vortioxetine affects connections among four large-scale functional brain networks, as well as the specific roles DTI-ALPS and PVS volume play in the therapeutic effects of vortioxetine on communication among these brain networks, mood, and cognitive performance in MDD, remains to be elucidated.

In this longitudinal-designed study, we investigated the effect of vortioxetine on brain glymphatic function (measured by DTI-ALPS and PVS volume), brain networks FC, mood, and cognition in individuals with MDD. We hypothesized that patients with MDD exhibited disrupted brain glymphatic function, brain networks FC, and cognitive functions. Vortioxetine would affect brain glymphatic function, brain networks FC, mood, and cognition in patients with MDD. Moreover, we also hypothesized that this change of brain glymphatic function would associate with the change in brain networks FC and cognitive performance in patients with MDD.

## 2. Methods

### 2.1. Participants

From April 2022 to October 2024, unmedicated patients with MDD in the acute phase were recruited from the First Affiliated Hospital of Jinan University, China. The diagnoses of MDD were based on the Diagnostic and Statistical Manual of Mental Disorders, Fifth Edition (DSM-V) by two psychiatrists (Yanbin Jia with 25 years and Shuming Zhong with 10 years of experience). The inclusion and exclusion criteria were comprehensively outlined in Supporting Information.

### 2.2. Study Design and Interventions

All participants underwent an initial clinical assessment and MRI scan at baseline. Patients with MDD were administered vortioxetine (20 mg/day) for an 8-week course of antidepressant therapy. Upon completion of the 8-week treatment period, these patients underwent a follow-up clinical assessment and MRI scan. Throughout the duration of the 8-week treatment, patients with MDD did not receive any additional medications.

### 2.3. Assessment of Mood Symptoms

Mood symptoms were assessed using the 24-item Hamilton Depression Rating Scale (HDRS-24) and Hamilton Anxiety Scale (HAMA). After treatment with vortioxetine, the changes in depressive and anxiety symptoms were assessed using the HDRS-24 score and HAMA score, respectively [27, 28]. Based on previous studies, remission is indicated by an HDRS-24 score that is 7 or below at posttreatment [26, 29].

### 2.4. Assessment of Cognition

Cognitive functions were performed utilizing the Measurement Consensus Cognitive Battery (MCCB; Chinese version), encompassing seven dimensions related to cognitive performance (Supporting Information).

### 2.5. MRI Data Acquisition

Multimodal imaging data (resting-state functional MRI, T1, DTI) were acquired utilizing the GE Discovery MR750 3.0 tesla system (8-channel phased array head coil; Supporting Information).

### 2.6. Data Preprocessing and Analysis of DTI-ALPS

**Figure 1:**
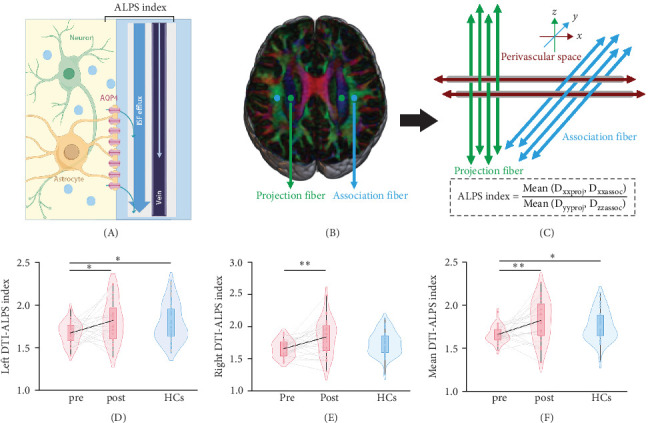
(A–F) Comparisons of the DTI-ALPS index between patients with MDD at pre- and posttreatment and HCs. *⁣*^*∗*^*p* < 0.05; *⁣*^*∗∗*^*p* < 0.01. DTI-ALPS, diffusion tensor imaging along the perivascular space; HCs, healthy controls; MDD, major depressive disorder.

DTI preprocessing was presented in Supporting Information. DTI-ALPS indexes for the whole brain were computed (Figure 1).

### 2.7. Data Preprocessing and Analysis of PVS Volume

The PVS analysis was conducted utilizing tools from the FSL 6.0.1 (https://fsl.fmrib.ox.ac.uk/fsl) [30], Advanced Normalization Tools (ANTs) software package [31], and Quantitative Imaging Toolkit (QIT; https://github.com/cabeen/qit/; Supporting Information). The volume of PVS was normalized to the total volume of the brain regions as the percent dilated PVS (pPVS) [32].

### 2.8. Data Preprocessing and Analysis of FC Among Four Networks

The resting-state functional MRI data preprocessing was presented in Supporting Information. A total of eight spherical regions of interest (ROIs) with a 5-mm radius as seeds were set for the four networks (DMN, ECN, SN, and AN) [26]. The specific coordinates of these seeds were as follow: the posterior cingulate cortex (PCC; MNI coordinates: ±7, −43, +33), dorsolateral prefrontal cortex (MNI coordinates: ±36, +27, +29), anterior insula (MNI coordinates: ±36, +18, +4), and subcallosal cingulate cortex (MNI coordinates: ±6, +24, −11; Figure S2).

For FC analysis, individual rs-FC maps were created by calculating the Pearson's correlation coefficients of time series between seeds and the whole brain voxel time series. For enhancing the normality, the Fisher's *r*-to-*z* transformation was subsequently performed to transform the subject-level correlation maps into *z*-value maps. Finally, we acquired eight *z*-score maps that depict the inherent FC of the ROIs across all subjects. All FC maps were smoothed using a 6 mm full width at half maximum Gaussian kernel.

### 2.9. Statistical Analysis

Statistical analysis was performed utilizing SPSS Statistics software. To assess demographic characteristics, clinical data (mood scales and cognitive functions), and the DTI-ALPS indexes between patients and HCs, independent-sample *t*-tests were employed for normally distributed variables, while the Mann–Whitney *U* test was utilized for skewed variables. A chi-squared test was conducted to evaluate the distribution of sex. A paired *t*-test was conducted to assess the changes in cognitive functions, DTI-ALPS index, and PVS volume in MDD after treatment. Multiple comparison correction was conducted utilizing the false discovery rate (FDR) method. Two-tailed *p* < 0.05 was considered statistically significant. The intraclass correlation coefficient (ICC) was performed to estimate interobserver agreement on the DTI-ALPS index measured by two radiologists.

For FC among four networks, a one-sample *t*-test was conducted on *z*-score maps for each ROI to demonstrate within-group FC spatial distribution for patients and HCs within a brain mask (*p* < 0.05, uncorrected). Two-sample *t*-tests and a pair *t*-test were subsequently conducted to evaluate the differences of the whole brain FC across regions between patients at pretreatment and HCs, patients at posttreatment and HCs, and patients at pretreatment and patients at posttreatment within the union mask of one-sample *t*-test results of both groups, by controlling for age, sex, years of education, and mean frame-wise displacement. The cluster-level multiple comparison correction was conducted using the Gaussian random field (GRF) theory (voxel-level *p* value  < 0.005; cluster-level *p* value  < 0.00625 [0.05/8]).

### 2.10. Correlation Analysis

Pearson's correlation analysis was used to explore the relationships among significant changed mood scales, cognitive functions, DTI-ALPS index, PVS volume, and FC in the group comparisons and paired *t*-tests. Pearson's correlation analyses were conducted in MDD group and HCs group, respectively.

## 3. Results

### 3.1. Participants

The demographic and clinical data of all participants are shown in Table 1. A total of 34 unmedicated patients with MDD in the acute phase and 41 HCs completed baseline and follow-up assessment of mood symptoms and cognitive functions. No significant differences were found in demographic data and YMRS score between MDD and HCs (*p* > 0.05). After treatment with vortioxetine, 23 patients (67.6%) achieved remission, 10 patients (29.4%) remained in the same stage, and only one patient (2.9%) went to worse. For MRI data, nine patients with MDD were excluded because of poor image quality, excessive head movement, or follow-up MRI scan refusal during either the baseline or follow-up MRI scans (Figure S1). Among these 25 patients with MDD and 41 HCs, no significant differences were found in demographic data and YMRS score (*p* > 0.05; Table S1).

### 3.2. Mood Scales and Cognitive Performance

At baseline, unmedicated patients with MDD in the acute phase displayed increased HDRS-24 (*p* < 0.001) and HAMA (*p* < 0.001) scores compared to HCs. For cognition, unmedicated MDD in the acute phase displayed decreased attention/vigilance (*p*=0.041), verbal learning (*p*=0.002), visual learning (*p*=0.026), social cognition (*p* < 0.001), and MCCB composite cognition score (*p* < 0.001) in the group comparison (Table 1).

After treatment with vortioxetine, the HDRS-24 (*p* < 0.001) and HAMA scores (*p* < 0.001) in the posttreatment significantly decreased compared to pretreatment in patients with MDD. For cognitive function, patients with MDD displayed significant increased attention/vigilance (*p*=0.022), verbal learning (*p*=0.040), visual learning (*p*=0.049), and MCCB composite cognition score (*p*=0.012) after treatment with vortioxetine (Table 2).

At follow-up, patients with MDD following treatment with vortioxetine displayed increased HDRS-24 (*p* < 0.001) and HAMA scores (*p* < 0.001) compared to HCs. For cognitive function, patients with MDD displayed decreased social cognition in the group comparison (*p* < 0.001; Table 1).

### 3.3. DTI-ALPS Index

The DTI-ALPS indexes measured by the two observers had excellent consistency (ICC = 0.83 [95% confidence interval (CI): 0.76, 0.89], ICC = 0.84 [95% CI: 0.77, 0.89], ICC = 0.88 [95% CI: 0.83, 0.92], respectively; Table S2).

At baseline, unmedicated patients with MDD displayed lower mean DTI-ALPS index (FDR-*p* < 0.05), lower left DTI-ALPS index (FDR-*p* < 0.05), and lower right DTI-ALPS index (*p* < 0.05, uncorrected) compared to HCs (Table 3).

After treatment with vortioxetine, the mean DTI-ALPS index, left DTI-ALPS index and right DTI-ALPS index in the posttreatment significantly increased compared to pretreatment in MDD (FDR-*p* < 0.05; Table 4).

At follow-up, no significant differences were found in the mean DTI-ALPS index, left DTI-ALPS index and right DTI-ALPS index between patients with MDD following treatment with vortioxetine and HCs (Table 3).

Moreover, we assessed DTI-ALPS indexes using corpus callosum (CC)-ROI *D*_*xx*_ values as nuisance covariates (Table S4). The results remain consistent with our primary findings (Tables S5 and S6).

### 3.4. PVS Volume

No significant differences were found in the pPVS volume within the white matter and subcortical regions when comparing unmedicated MDD in the acute phase to HCs at both baseline and follow-up (Table 3). After treatment with vortioxetine, no significant changes were found in the pPVS volume within the white matter and subcortical regions in unmedicated MDD in the acute phase (Table 4).

### 3.5. FC Among Four Networks

A one-sample *t*-test identified within-group FC patterns were presented in Figure S3 for each brain regional seed (*p* < 0.05).

**Figure 2:**
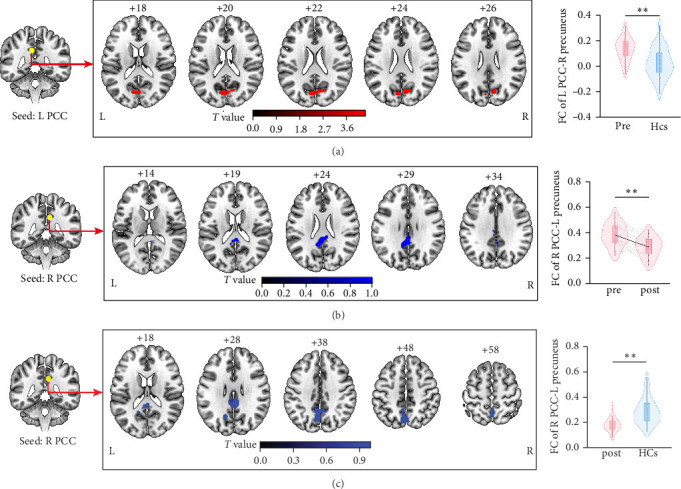
The significant whole brain FC differences among four networks. (A) FC differences for left PCC seed between patients with MDD and HCs at pretreatment. (B) FC differences for right PCC seed between patients with MDD and HCs at posttreatment. (C) FC differences for right PCC seed in patients with MDD after treatment with vortioxetine. The color bar indicates the *T* values from two-sample *t*-tests and a pair *t*-test. FC, functional connectivity; HCs, healthy controls; L/R, left/right hemisphere; MDD, major depressive disorder; PCC, posterior cingulate cortex. *⁣*^*∗∗*^*p* < 0.01.

As for the PCC seed FC analysis for probing the DMN, unmedicated patients with MDD in the acute phase displayed increased left PCC-bilateral precuneus FC compared to HCs (voxel *p* value  < 0.005; cluster significance: *p* < 0.00625 [0.05/8], GRF corrected; Figure 2A, Table S3) at baseline. No significant hypo- or hyperconnectivity for the SN, ECN, and AN was found when comparing MDD and HCs at baseline.

After treatment with vortioxetine, the right PCC-bilateral precuneus FC in the posttreatment significantly decreased compared to pretreatment in patients with MDD (voxel *p* value  < 0.005; cluster significance: *p* < 0.00625 [0.05/8], GRF corrected; Figure 2B, Table S3). No significant hypo- or hyperconnectivity for the SN, ECN, and AN was found in MDD from baseline to posttreatment.

At follow-up, patients with MDD following treatment with vortioxetine displayed decreased right PCC-bilateral precuneus FC compared to HCs (voxel *p* value  < 0.005; cluster significance: *p* < 0.00625 [0.05/8], GRF corrected; Figure 2C, Table S3). No significant hypo- or hyperconnectivity for the SN, ECN, and AN was found when comparing MDD and HCs at follow-up.

### 3.6. Correlation Analysis

**Figure 3:**
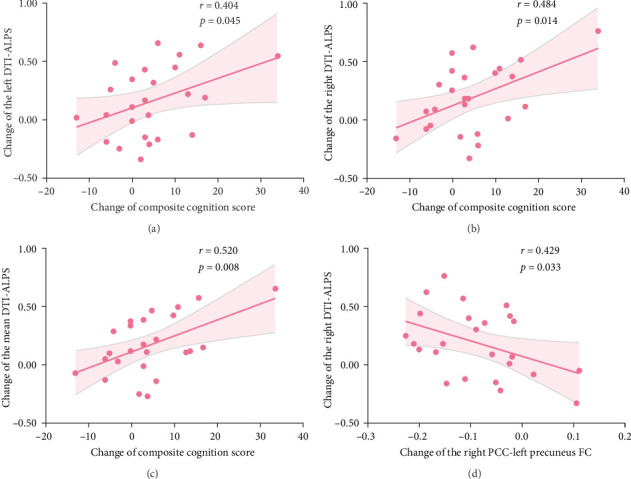
(A–D) Correlation analysis in patients with MDD. DTI-ALPS, diffusion tensor imaging along the perivascular space; FC, functional connectivity; MDD, major depressive disorder; PCC, posterior cingulate cortex.

The change of composite cognition score for global cognition was positively correlated with the change of the left DTI-ALPS index (*r* = 0.404, *p*=0.045; Figure 3A), right DTI-ALPS index (*r* = 0.484, *p*=0.014; Figure 3B), and mean DTI-ALPS index (*r* = 0.520, *p*=0.008) in MDD (Figure 3C), following treatment with vortioxetine. The change of the right PCC-bilateral precuneus FC was negatively correlated with the change of the right DTI-ALPS index in MDD (*r* = −0.429, *p*=0.033; Figure 3D), following treatment with vortioxetine. The change of diffusivity along the *x*-axis in the left association fibers area (*D*_*xx*assoc_) was positively correlated with the change of verbal learning score (*r* = 0.412, *p*=0.041; Figure S4A) and composite cognition score (*r* = 0.472, *p*=0.017) in MDD (Figure S4B), following treatment with vortioxetine. No significant correlation was observed between mood scales, cognitive functions, FC, and DTI-ALPS index in the MDD group at baseline and follow-up.

No significant correlation was observed between mood scales, cognitive functions, FC, and DTI-ALPS index in the HCs.

## 4. Discussion

This is the first study to explore the therapeutic effects of an 8-week vortioxetine treatment on brain glymphatic function, FC among large-scale functional brain networks, mood, and cognition in MDD. The main findings were as follows: (1) Treatment with vortioxetine significantly improved impaired brain glymphatic function, as indicated by the DTI-ALPS index, in MDD. (2) Vortioxetine modulated abnormal DMN connections in MDD. (3) Vortioxetine significantly improved impaired mood and cognitive performance in MDD. (4) The increase of DTI-ALPS index was associated with the improvement in cognition and DMN FC in MDD following vortioxetine treatment. These findings could provide new clues to understand the neuropathological mechanisms underlying the antidepressant effects of vortioxetine.

In the current study, we found that unmedicated adult non-elderly patients with MDD showed lower DTI-ALPS index compared to HCs, suggesting impaired brain glymphatic system function in MDD. Bidirectional effect between neuroinflammation and brain glymphatic system function has been identified in MDD. Neuroinflammation may impair astrocyte function, disrupt homeostasis, and thus, contribute to the development of MDD [15, 16]. Activated microglia release inflammatory factors that may influence the clearance processes of the glymphatic system, which might, in turn, modulate neuroinflammation [15]. The proper functioning of the glymphatic system is contingent upon the polarized distribution of aquaporin 4 (AQP4), a water channel that facilitates the movement of water across the cell membrane [6]. A previous animal study demonstrated that depressed mice exhibited significant neuroinflammation, reduced arterial pulsation and compliance in the brain, and depolarized expression of AQP4, suggesting the presence of glymphatic dysfunction and the accumulation of amyloid Aβ42 in the brain parenchyma [3]. More importantly, we found that treatment with vortioxetine resulted in a significant increase in the DTI-ALPS index, indicating a restoration of glymphatic system function following vortioxetine administration. It had been previously reported that treatment with the antidepressant fluoxetine can reverse the slowed clearance of the glymphatic system and the accumulation of Aβ42 in the brain [33]. A previous preclinical study revealed that dietary supplementation with polyunsaturated fatty acids alleviated rescued depression-like behaviors, reduced neuroinflammation, and improved glymphatic system dysfunction in depression mice [3]. Another study demonstrated that melatonin rescued depressive symptoms in mouse model of depression by modulating Per2 circadian protein expression, maintaining astrocytic AQP4 polarization circadian rhythm, and restoring glymphatic function [34]. Intriguingly, no differences were found in the PVS volume between unmedicated MDD in the acute phase and HCs. Glymphatic dysfunction would slow down the clearance of metabolic waste products in the brain, and the accumulation of metabolic waste would lead to PVS enlargement [9, 35]. It is widely accepted that changes in brain function typically precede structural changes [36]. It was speculated that patients with MDD exhibited impaired glymphatic function, measured by DTI-ALPS, but not structural PVS enlargement during the acute phase of the disorder. In short, our findings suggested that administration with vortioxetine can rescue impaired brain glymphatic system function in the acute phase of MDD.

We found patients with MDD exhibited increased FC between the PCC and precuneus compared to HCs. Treatment with vortioxetine resulted in a reduction of this elevated pattern of FC between the PCC and precuneus. The PCC and precuneus are critical nodes within the DMN, a large-scale functional brain network that is believed to support internally-oriented and self-referential thought [21]. In the realm of clinical neuroscience related to MDD, the DMN is the neural network that has garnered the most focus [37]. Consistent with our findings, a previous meta-analysis of FC identified that MDD was characterized by hyperconnectivity within the DMN [21]. Prior studies using task-based fMRI to explore neural activity in the context of the antidepressant therapeutic effect have reported a connection between treatment response and decreased fMRI activation in DMN-related regions [38–40]. A hyperactive response in the DMN is thought to represent an effortful compensatory mechanism during maintained performance in the *N*-back task in individuals with MDD [40]. Vortioxetine can enhance the capacity to attenuate the overactivation of the DMN during executive function tasks [40]. Similarly, a multicenter large-scale study also proposed that FC within the DMN significantly decreased in MDD after antidepressant medication treatment [25]. Taken together, it was speculated that the overactivation of the DMN observed before treatment in patients with MDD might involve a compensatory mechanism that can be normalized by treatment with vortioxetine. Moreover, we found that the change in FC between the right PCC and bilateral precuneus was correlated with the change in the right DTI-ALPS index in MDD, following vortioxetine treatment. Current evidence indicated that the glymphatic system contributes to protecting neural interactions in the neural network [41]. Glymphatic system dysfunction may adversely affect the neural activity of the early-stage brain network by facilitating the accumulation of toxic proteins, thereby disrupting functional interactions across widespread brain regions [41]. To sum up, our findings added crucial evidence that as the glymphatic system improves, there is a corresponding improvement of the communication within the DMN among patients with MDD following vortioxetine treatment.

In addition, we found vortioxetine significantly improved impaired attention/vigilance, verbal learning, visual learning, and global cognition in patients with MDD. Notably, we identified that the increase of global cognition was correlated with the increase of the mean DTI-ALPS index in MDD after vortioxetine treatment. Vortioxetine, a novel treatment for depression, exhibits multimodal functional effects through the modulation of various neurotransmitter systems [40]. Compared with other antidepressants, vortioxetine has a distinctive pharmacological profile that may improve the impaired cognitive performance of patients with MDD [4, 5, 42]. Researchers found that worse cognitive functions was correlated with lower DTI-ALPS index in neurological and neurodegenerative disorders [9, 43–45], suggesting an independent key role of DTI-ALPS index in cognitive decline [44]. Preclinical evidence on mouse suggested that although treatment with an antidepressant alleviates depression-like behavioral symptoms, cognitive deficits persist unless dysfunction of the glymphatic system is addressed [3]. The study indicated that these benefits were likely achieved through the restauration of glymphatic system function [3]. Together, our preliminary findings suggested a close association between the enhanced global cognition and the improved glymphatic system function in MDD after vortioxetine treatment.

Several limitations must be acknowledged. First of all, this analysis was conducted at a single center with a relatively small number of participants. Second, this noninvasive methodology cannot be considered a direct replacement for the gold standard in evaluating glymphatic function, despite its provision of valuable insights into the underlying neuropathological mechanisms of MDD. It would be advantageous to validate the current findings using invasive gold standard approaches. Third, emerging tools (e.g., artRepair) can detect artifacts in resting-state fMRI images, allowing retention of subjects with clean images despite excessive movement beyond standard thresholds. Moreover, the correlation analysis in the study was exploratory, as they did not survive after multiple comparison corrections. Finally, incorporating a placebo group into the design of randomized controlled trial would be beneficial in the future.

## 5. Conclusions

In conclusion, the current study offered empirical evidence supporting the efficacy of vortioxetine in modulating impaired brain glymphatic function in patients with MDD. Moreover, the improvement in glymphatic function was associated with the enhancement in communication within the DMN and cognitive functions.

## Figures and Tables

**Table 1:** Demographic and clinical data for participates.

Variable	MDD	HCs	*p*-Value
Demographic			
Number of participants	34	41	N/A
Sex (male/female)	9/25	16/25	0.251^b^
Age (years)	25.26 (6.34)	23.10 (3.32)	0.078^a^
Education (years)	15.47 (2.62)	16.05 (2.51)	0.333^a^
Number of episodes	2.30 (1.70)	—	—
Age of onset (years)	20.94 (6.31)	—	—
Duration of illness (months)	23.71 (41.03)	—	—
Mood scales			
Baseline HDRS-24 score	25.27 (5.14)	2.15 (2.17)	<0.001^a^*⁣*^*∗*^
Baseline HAMA score	13.24 (5.27)	1.64 (2.44)	<0.001^a^*⁣*^*∗*^
Baseline YMRS score	1.85 (2.67)	0.92 (1.89)	0.145^a^
Posttreatment HDRS-24 score	6.79 (5.10)	2.15 (2.17)	<0.001^a^*⁣*^*∗*^
Posttreatment HAMA score	4.24 (3.46)	1.64 (2.44)	<0.001^a^*⁣*^*∗*^
MCCB			
Baseline processing speed score	46.88 (7.83)	49.73 (6.52)	0.089^a^
Baseline attention/vigilance score	46.64 (7.93)	50.20 (6.90)	0.041^a^*⁣*^*∗*^
Baseline working memory score	48.33 (9.27)	47.93 (8.40)	0.843^a^
Baseline verbal learning score	43.61 (9.73)	50.41 (8.68)	0.002^a^*⁣*^*∗*^
Baseline visual learning score	49.48 (6.45)	52.78 (6.07)	0.026^a^*⁣*^*∗*^
Baseline reasoning problem-solving score	52.58 (8.12)	51.24 (7.87)	0.474^a^
Baseline social cognition score	42.03 (11.84)	54.15 (11.73)	<0.001^a^*⁣*^*∗*^
Baseline overall composite score	44.55 (7.34)	51.13 (5.04)	<0.001 ^a^*⁣*^*∗*^
Posttreatment processing speed score	49.45 (7.55)	49.73 (6.52)	0.865
Posttreatment attention/vigilance score	49.33 (7.96)	50.20 (6.90)	0.619
Posttreatment working memory score	48.00 (9.78)	47.93 (8.40)	0.972
Posttreatment verbal learning score	47.45 (7.96)	50.41 (8.68)	0.131
Posttreatment visual learning score	52.52 (6.06)	52.78 (6.07)	0.851
Posttreatment reasoning problem-solving score	53.52 (8.34)	51.24 (7.87)	0.230
Posttreatment social cognition score	42.61 (10.24)	54.15 (11.73)	<0.001^a^*⁣*^*∗*^
Posttreatment overall composite score	48.36 (7.20)	51.13 (5.04)	0.056

*Note:* Mean (S.D.) are reported. MCCB, Chinese version of Measurement Consensus Cognitive Battery.

Abbreviations: HAMA, Hamilton Anxiety Scale; HCs, healthy controls; HDRS, Hamilton Depression Rating Scale; MDD, major depressive disorder.

^a^The *p* values were obtained by independent-sample *t*-tests.

^b^The *p* value for gender distribution was obtained by chi-square test.

*⁣* ^*∗*^ *p* < 0.05.

**Table 2:** Changes of the mood and cognition after an 8-week treatment with vortioxetine in MDD.

Variable	Pretreatment	Posttreatment	*p*
Mood scales			
HDRS-24 score	25.27 (5.14)	6.79 (5.10)	<0.001^a^*⁣*^*∗*^
HAMA score	13.24 (5.27)	4.24 (3.46)	<0.001^a^*⁣*^*∗*^
MCCB			
Processing speed score	46.88 (7.83)	49.45 (7.55)	0.052^a^
Attention/vigilance score	46.64 (7.93)	49.33 (7.96)	0.022^a^*⁣*^*∗*^
Working memory score	48.33 (9.27)	48.00 (9.78)	0.791^a^
Verbal learning score	43.61 (9.73)	47.45 (7.96)	0.040^a^*⁣*^*∗*^
Visual learning score	49.48 (6.45)	52.52 (6.06)	0.051^a^
Reasoning problem-solving score	52.58 (8.12)	53.52 (8.34)	0.429 ^a^
Social cognition score	42.03 (11.84)	42.61 (10.24)	0.788^a^
Overall composite score	44.55 (7.34)	48.36 (7.20)	0.012^a^*⁣*^*∗*^

*Note:* Mean (S.D.) are reported. MCCB, Chinese version of Measurement Consensus Cognitive Battery.

Abbreviations: HAMA, Hamilton Anxiety Scale; HDRS, Hamilton Depression Rating Scale; MDD, major depressive disorder.

^a^The *p* values were obtained by paired-sample *t*-tests.

*⁣* ^*∗*^ *p* < 0.05.

**Table 3:** Differences of glymphatic system function between MDD and HCs.

Variable	MDD (*n* = 25)	HCs (*n* = 41)	*p*	FDR-p
Baseline				
DTI-ALPS				
Left hemisphere				
Association fiber				
*D*_*xx*_ (×10^−3^ mm^2^/s)	0.74 (0.08)	0.74 (0.10)	0.706	0.776
*D*_*zz*_ (×10^−3^ mm^2^/s)	0.38 (0.06)	0.35 (0.05)	0.105	0.144
Projection fiber				
*D*_*xx*_ (×10^−3^ mm^2^/s)	0.65 (0.06)	0.67 (0.07)	0.098	0.144
*D*_*yy*_ (×10^−3^ mm^2^/s)	0.45 (0.04)	0.45 (0.05)	0.881	0.881
Right hemisphere				
Association fiber				
*D*_*xx*_ (×10^−3^ mm^2^/s)	0.72 (0.07)	0.76 (0.09)	0.044*⁣*^*∗*^	0.081
*D*_*zz*_ (×10^−3^ mm^2^/s)	0.39 (0.05)	0.37 (0.06)	0.127	0.155
Projection fiber				
*D*_*xx*_ (×10^−3^ mm^2^/s)	0.66 (0.06)	0.69 (0.06)	0.038*⁣*^*∗*^	0.081
*D*_*yy*_ (×10^−3^ mm^2^/s)	0.45 (0.05)	0.48 (0.05)	0.031*⁣*^*∗*^	0.081
Left DTI-ALPS index	1.67 (0.13)	1.78 (0.22)	0.007*⁣*^*∗*^	0.039*⁣*^*∗*^
Right DTI-ALPS index	1.65 (0.13)	1.73 (0.18)	0.041*⁣*^*∗*^	0.081
Mean DTI-ALPS index	1.66 (0.09)	1.76 (0.18)	0.003*⁣*^*∗*^	0.033*⁣*^*∗*^
PVS volume				
White matter	0.195 [0.183, 0.212]	0.196 [0.184, 0.211]	0.798	N/A
Subcortical regions	0.154 [0.147, 0.164]	0.153 [0.144, 0.162]	0.421	N/A
8-week follow-up				
DTI-ALPS				
Left hemisphere				
Association fiber				
*D*_*xx*_ (×10^−3^ mm^2^/s)	0.80 (0.10)	0.74 (0.10)	0.012*⁣*^*∗*^	0.044*⁣*^*∗*^
*D*_*zz*_ (×10^−3^ mm^2^/s)	0.39 (0.05)	0.35 (0.05)	0.009*⁣*^*∗*^	0.044*⁣*^*∗*^
Projection fiber				
*D*_*xx*_ (×10^−3^ mm^2^/s)	0.67 (0.06)	0.67 (0.07)	0.478	0.621
*D*_*yy*_ (×10^−3^ mm^2^/s)	0.45 (0.06)	0.45 (0.05)	0.993	0.993
Right hemisphere				
Association fiber				
*D*_*xx*_ (×10^−3^ mm^2^/s)	0.78 (0.14)	0.76 (0.09)	0.508	0.621
*D*_*zz*_ (×10^−3^ mm^2^/s)	0.36 (0.06)	0.37 (0.06)	0.386	0.607
Projection fiber				
*D*_*xx*_ (×10^−3^ mm^2^/s)	0.66 (0.06)	0.69 (0.06)	0.052	0.143
*D*_*yy*_ (×10^−3^ mm^2^/s)	0.44 (0.06)	0.48 (0.05)	0.009*⁣*^*∗*^	0.044*⁣*^*∗*^
Left DTI-ALPS index	1.81 (0.25)	1.78 (0.22)	0.603	0.663
Right DTI-ALPS index	1.83 (0.28)	1.73 (0.18)	0.126	0.277
Mean DTI-ALPS index	1.82 (0.24)	1.76 (0.18)	0.210	0.385
PVS volume				
White matter	0.193 [0.180, 0.210]	0.196 [0.184, 0.211]	0.690	N/A
Subcortical regions	0.154 [0.145, 0.162]	0.153 [0.144, 0.162]	0.857	N/A

*Note:* Mean (S.D.) or median [interquartile range] are reported.

Abbreviations: DTI-ALPS, diffusion tensor imaging along the perivascular space; *D*_*xx*_, diffusivity along the *x*-axis; *D*_*yy*_, diffusivity along the *y*-axis; *D*_*zz*_, diffusivity along the *z*-axis; FDR, false discovery rate; HCs, healthy controls; MDD, major depressive disorder; PVS, perivascular space.

*⁣* ^*∗*^ *p* < 0.05.

**Table 4:** Changes of glymphatic system function after an 8-week treatment with vortioxetine in MDD.

Variable	Pretreatment	Posttreatment	*p*	FDR-p
DTI-ALPS				
Left hemisphere				
Association fiber				
*D*_*xx*_ (×10^−3^ mm^2^/s)	0.74 (0.08)	0.80 (0.10)	0.003*⁣*^*∗*^	0.008*⁣*^*∗*^
*D*_*zz*_ (×10^−3^ mm^2^/s)	0.38 (0.06)	0.39 (0.05)	0.542	0.663
Projection fiber				
*D*_*xx*_ (×10^−3^ mm^2^/s)	0.65 (0.06)	0.67 (0.06)	0.355	0.558
*D*_*yy*_ (×10^−3^ mm^2^/s)	0.45 (0.04)	0.45 (0.06)	0.811	0.837
Right hemisphere				
Association fiber				
*D*_*xx*_ (×10^−3^ mm^2^/s)	0.72 (0.07)	0.78 (0.14)	0.076	0.140
*D*_*zz*_ (×10^−3^ mm^2^/s)	0.39 (0.05)	0.36 (0.06)	<0.001*⁣*^*∗*^	0.004*⁣*^*∗*^
Projection fiber				
*D*_*xx*_ (×10^−3^ mm^2^/s)	0.66 (0.06)	0.66 (0.06)	0.416	0.571
*D*_*yy*_ (×10^−3^ mm^2^/s)	0.45 (0.05)	0.44 (0.06)	0.837	0.837
Left DTI-ALPS index	1.67 (0.13)	1.81 (0.25)	0.013*⁣*^*∗*^	0.027*⁣*^*∗*^
Right DTI-ALPS index	1.65 (0.13)	1.83 (0.28)	0.003*⁣*^*∗*^	0.008*⁣*^*∗*^
Mean DTI-ALPS index	1.66 (0.09)	1.82 (0.24)	0.002*⁣*^*∗*^	0.008*⁣*^*∗*^
PVS volume				
White matter	0.195 [0.183, 0.212]	0.193 [0.180, 0.210]	0.925	N/A
Subcortical regions	0.154 [0.147, 0.164]	0.154 [0.145, 0.162]	0.614	N/A

*Note:* Mean (S.D.) or median [interquartile range] are reported.

Abbreviations: DTI-ALPS, diffusion tensor imaging along the perivascular space; *D*_*xx*_, diffusivity along the *x*-axis; *D*_*yy*_, diffusivity along the *y*-axis; *D*_*zz*_, diffusivity along the *z*-axis; FDR, false discovery rate; MDD, major depressive disorder; PVS, perivascular space.

*⁣* ^*∗*^ *p* < 0.05.

## Data Availability

The data that support the findings of this study are available upon request from the corresponding author. The data are not publicly available due to privacy or ethical restrictions.

## Supporting Information

Supporting Information Figure S1. The flowchart of participants through the study. Figure S2. Eight seeds for the four networks in the bilateral hemisphere. Figure S3. One-sample *t*-test within-group FC patterns. Figure S4. Correlation analysis for diffusivity values in the *x*, *y*, and *z*-directions for projection fibers and association fibers in MDD. Table S1. Demographic for participants in MRI analysis. Table S2. Interobserver agreement on diffusivities and DTI-ALPS indexes. Table S3. The significant FC difference in group comparisons. Table S4. Differences of *D*_*xx*_ index in the corpus callosum. Table S5. Differences of DTI-ALPS indexes between MDD and HCs with *D*_*xx*_ index in the corpus callosum included as a nuisance covariate. Table S6. Changes of DTI-ALPS indexes after treatment with vortioxetine in MDD with *D*_*xx*_ index in the corpus callosum included as a nuisance covariate.
