# Supplementary material for: Vortioxetine Improves Brain Glymphatic System Function, Functional Connectivity, and Cognitive Functions in Major Depressive Disorder
Source: Depress Anxiety. 2025 Aug 30;2025:1990117. doi: 10.1155/da/1990117 (PMC12413946; doi:10.1155/da/1990117)
Supplement: Supporting Information — Figure S1. The flowchart of participants through the study. Figure S2. Eight seeds for the four networks in the bilateral hemisphere. Figure S3. One-sample t-test within-group FC patterns. Figure S4. Correlation analysis for diffusivity values in the x, y, and z-directions for projection fibers and association fibers in MDD. Table S1. Demographic for participants in MRI analysis. Table S2. Interobserver agreement on diffusivities and DTI-ALPS indexes. Table S3. The significant FC difference in group comparisons. Table S4. Differences of Dxx index in the corpus callosum. Table S5. Differences of DTI-ALPS indexes between MDD and HCs with Dxx index in the corpus callosum included as a nuisance covariate. Table S6. Changes of DTI-ALPS indexes after treatment with vortioxetine in MDD with Dxx index in the corpus callosum included as a nuisance covariate. [file 1990117.f1.docx]

***Supplementary Materials***

**Vortioxetine improves glymphatic system function, brain functional connectivity, and cognition in major depressive disorder**

**Graphical abstract**

**
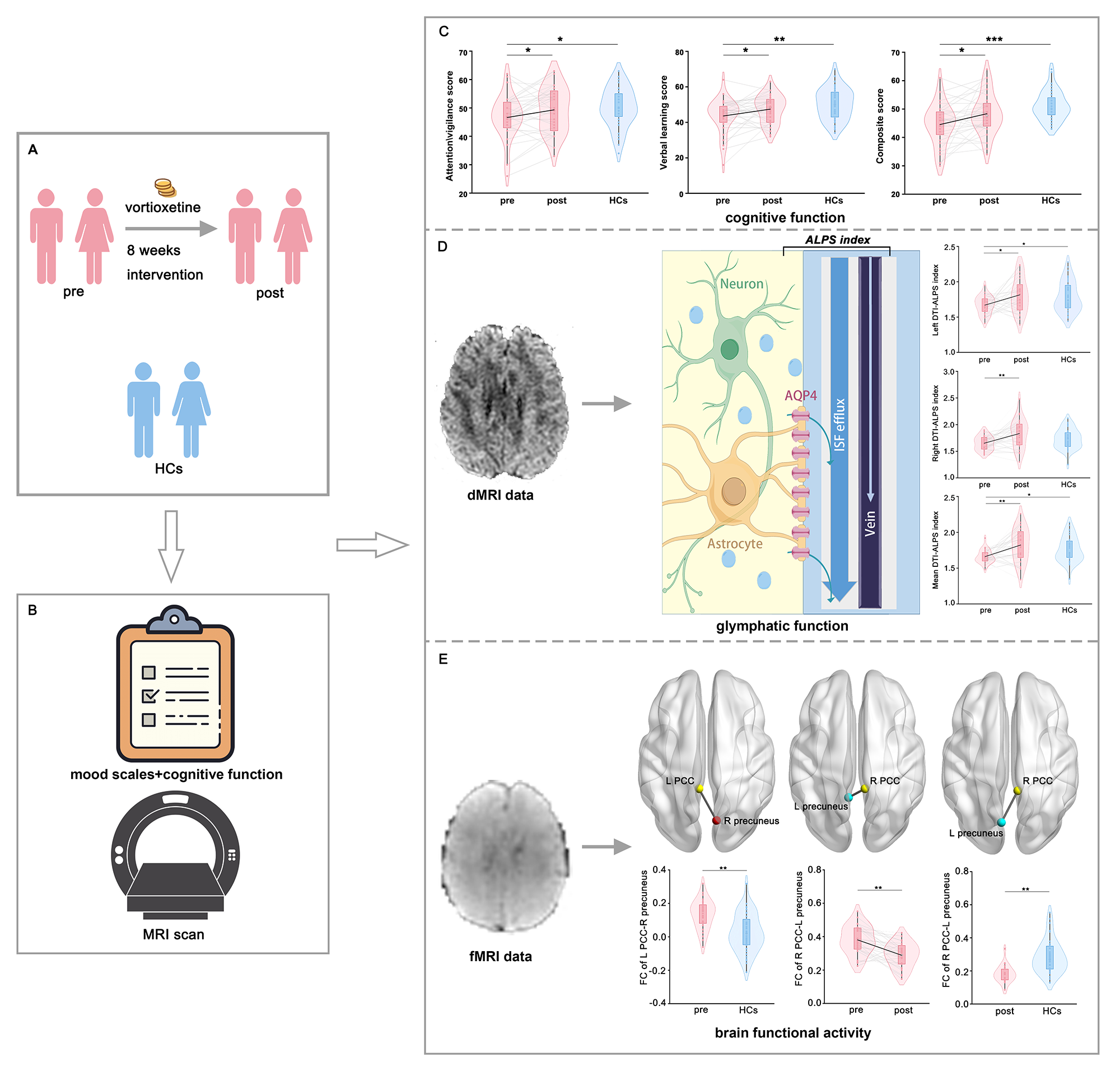
**

**Methods**

**Participants**

The inclusion criteria for patients with MDD were as follows: (1) the 24-item Hamilton Depression Rating Scale (HDRS-24) score > 21 and the Young Mania Rating Scale (YMRS) score < 7; (2) aged 18-45 years; (3) right-handed, Han nationality. The exclusion criteria for MDD were as follow: (1) presence of other Axis-I psychiatric disorders; (2) any major concurrent physical illness; (3) lactating or pregnant; (4). The inclusion criteria for HCs were as follows: (1) no history of any psychiatric and major physical illness; (2) not being lactating or pregnant.

The study was approved by the ethics committee of the First Affiliated Hospital of Jinan University (Guangzhou, China). Prior to their participation, all subjects involved provided written informed consent after receiving comprehensive written and verbal explanations regarding the study.

**MRI Data Acquisition**

Participants were scanned in a supine, head-forward position, and placed cushions symmetrically on both sides of the head to reduce movement. Before the scan, participants were instructed to relax and close their eyes without falling asleep repeatedly; after the experiment, only participants who confirmed that they were not asleep were included in the study; otherwise, he / she will be excluded. Each participant completed MRI scanning before and after 8 weeks of treatment. The rs-fMRI data were acquired using a gradient-echo echo-planar imaging (EPI) sequence with time repetition (TR) = 2000 ms, the other parameters were as the following: time echo (TE) = 25 ms, matrix = 64 × 64, voxel size = 3.75 × 3.75 × 3mm³, field of view (FOV) = 240 × 240 mm, flip angle = 90°, 35 axial slices covering the whole-brain, slice thickness/gap = 3.0/1.0 mm, and 210 volumes acquired in 7 minutes. Additionally, a three-dimensional brain volume imaging (3D-BRAVO) sequence with the following parameters: TR/TE = 8.2/3.2 ms, slice thickness/gap = 1.0/0 mm, bandwidth = 31.25 Hz, flip angle = 12°, FOV = 240 × 240 mm, matrix = 256 × 256, NEX = 1, and acquisition time = 3 min 45 s. The diffusion tensor imaging (DTI) data were obtained using a single-shot multi-slice spin-echo diffusion-weighted EPI sequence with the following parameters: TR = 8000 ms; TE = 68 ms; FOV = 256 × 256 mm^2^; data matrix = 128 × 128; slice thickness = 2mm, without inter-slice gap; flip angle = 90°; 30 diffusion-sensitive directions with *b* = 1000 mm^2^/s; 5 volumes with *b*_0_ = 0 mm^2^/s, and 75 axial interleaved slices covering the whole brain; and acquisition time = 4 min 48 s. Axial fast spine echo T2-weighted and T1-weighted fluid attenuation inversion recovery MRI examination images were collected to confirm the absence of any brain structural abnormality. Two experienced radiologists (Z.G. and Z.Q., with 6 and 7 years of experience in neuroimaging, respectively) confirmed that there were no brain structural abnormalities.

**Assessment of Cognitions**

Participants' cognitive function was assessed using the MATRICS Consensus Cognitive Battery (MCCB) [^1^](#_ENREF_1). This instrument assesses seven cognitive-associated dimensions using 10 subscales, including (i) processing speed through the following three subtests: Trail Making Test-A (TMT-A) [^2^](#_ENREF_2), Category Fluency (Animal Naming), and the Brief Assessment of Cognition in Schizophrenia-Symbol Coding (BACS-SC); (ii) attention/vigilance through the Continuous Performance Test-Identical Paris (CPT-IP); (iii) WM through the Wechsler Memory Scale-Third Edition Spatial Span (WMS-III SS); (iv) verbal learning through the Hopkins Verbal Learning Test-Revised (HVLT-R); (v) visual learning through the Brief Visuospatial Memory Test-Moderated (BVMT-R); (vi) reasoning and problem-solving ability through the Neuropsychological Assessment Battery: Maze (NABM); and (vii) social cognition through the Mayer-Salovey-Caruso Emotional Intelligence Test. Higher MCCB test scores indicated better cognitive function.

**Data Preprocessing and Analysis of DTI-ALPS**

DTI preprocessing was performed using tools from the FMRIB Software Library (FSL) 6.0.1 (<https://fsl.fmrib.ox.ac.uk/fsl/>). DTI data were first corrected for subject movement and eddy current–induced distortion using the “eddy_correct” function of FSL. The DTI data were then skull-stripped using the Brain Extraction Tool and registered to standard space. Diffusion tensor maps were computed using the “DTIFIT” function of FSL. Color-coded fractional anisotropy maps and diffusivity maps in directions of the x-, y-, and z-axes (D_xx_, D_yy_, D_zz_) were generated. Afterwards, two neuroradiologists (5 and 11 years of experience) independently measured the regions of interest (ROI) based on the following diffusive parameters, blinded to clinical and demographic information. The corona radiata projection and the superior longitudinal fasciculus were used to represent the projection and association fibers, respectively, for the ALPS index calculation [^3^](#_ENREF_3). Diffusivity values were registered in the x, y, and z directions for projection fibers and association fibers, labeled as D_xxproj_, D_yyproj_, D_zzproj_, D_xxassoc_, D_yyassoc_, and D_zzassoc_. All of these metrics were measured after placing a 2.5-mm radius spherical ROI in the axial slices above the bilateral ventricles in both hemispheres according to the previous study [^4^](#_ENREF_4)^,^[^5^](#_ENREF_5). The ALPS index was computed as ([mean(D_xxproj_, D_xxassoc_)/mean(D_yyproj_, D_zzassoc_)]) [^3^](#_ENREF_3). Additionally, crossing fibers coming from corpus callosum (CC) might contribute to D_xx_ changes in DTI-ALPS ROIs. To evaluate this possibility, a matched ROI was placed in the CC at the same level than DTI-ALPs ROIs (Tables S4-S6).

**Data Preprocessing and Analysis of PVS Volume**

The following steps were undertaken: As a first step, each subject was examined for scanner artifacts and gross anatomical abnormalities. Secondly, bias-corrected T1 images were segmented using an automated algorithm. Thirdly, excess tissue was removed from the image, and the skull was scraped off. Fourthly, images were registered to Montreal Neurological Institute (MNI) 152 space using FSL’s FNIRT. Fifthly, the parcellated brain (including white matter, and subcortical nuclei) was served as a mask for PVS quantification. Sixthly, the Frangi filter [^6^](#_ENREF_6) was applied to T1 images using QIT. The seventh step consisted of applying the optimal threshold of 2.3 to T1 images in order to generate a PVS map [^7^](#_ENREF_7). Finally, the volume of PVS was normalized to the total volume of the brain regions as the percent dilated PVS (pPVS) [^8^](#_ENREF_8). The subcortical regions include bilateral amygdala, basal ganglia, hippocampus, and thalamus.

**Data Preprocessing and** **Analysis of FC among four networks**

The resting-state functional MRI data preprocessing was conducted using the the Statistical Parametric Mapping (SPM12) and the Data Processing Assistant for Resting-State fMRI (DPABI_V3.0). A consistent longitudinal magnetization was maintained by excluding the initial 10 images in the rs-fMRI dataset. Following slice-time correction, the remaining 200 images were realigned to the initial image to correct for TR head motion. With the adjustment of this realignment, a document of head movement could be obtained during the rs-fMRI scan. Subject whose displacement exceeds 2 mm in any direction, 2° in angular motion, or a mean frame-wise displacement (FD) of 0.2mm will be excluded [^9^](#_ENREF_9). Using a segmentation toolbox, the T1 structural images were classified as gray matter, white matter, and cerebrospinal fluid. A template specific to the study was generated using the DARTEL toolbox to ensure precise normalization. The structural images were used to co-register and transform the resting-state functional images into standard Montreal Neurological Institute (MNI) space. The voxel size was adjusted to a resolution of 3×3×3mm^3^ by reslicing. The data was detrended and then filtered between 0.01Hz to 0.1 Hz using a band-pass filter. The time course of each voxel was refined by eliminating extraneous variables and their temporal derivatives, such as the brain global mean, white matter, cerebrospinal fluid signals, and the Friston-24 head motion parameters (consisting of 6 head motion parameters from the previous time point and the corresponding 12 squared items) [^10^](#_ENREF_10)^,^[^11^](#_ENREF_11). A 6mm full width at half maximum (FWHM) Gaussian kernel was used for smoothing.


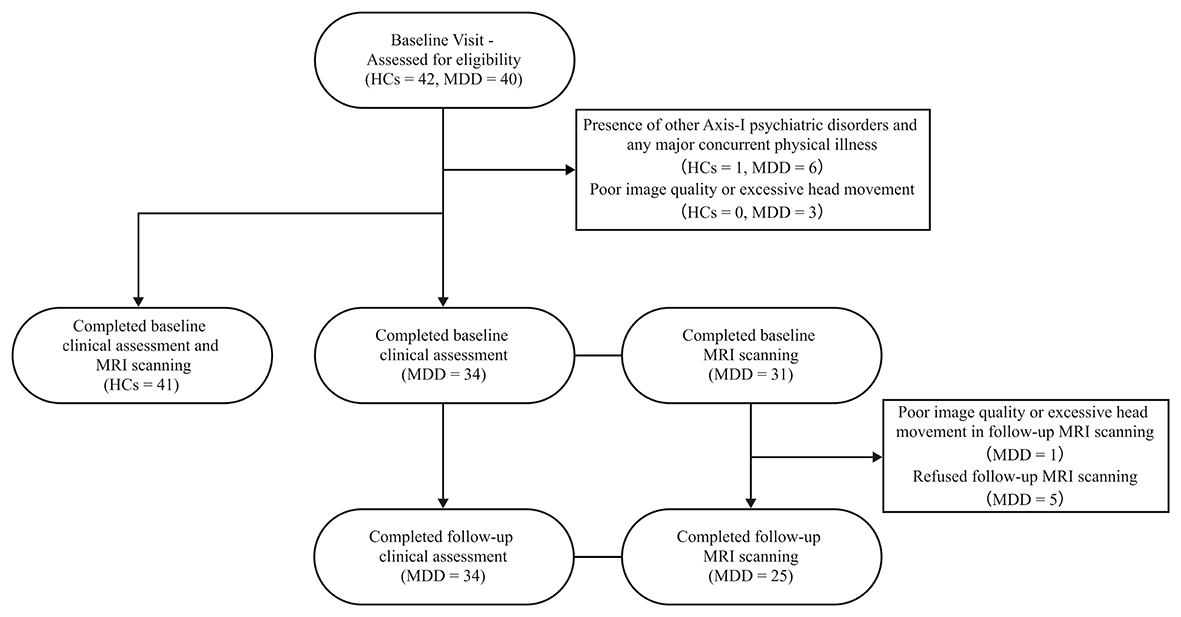


**Figure S1.** The flowchart of participants through the study. MDD, major depressive disorder; HCs. healthy controls.

**
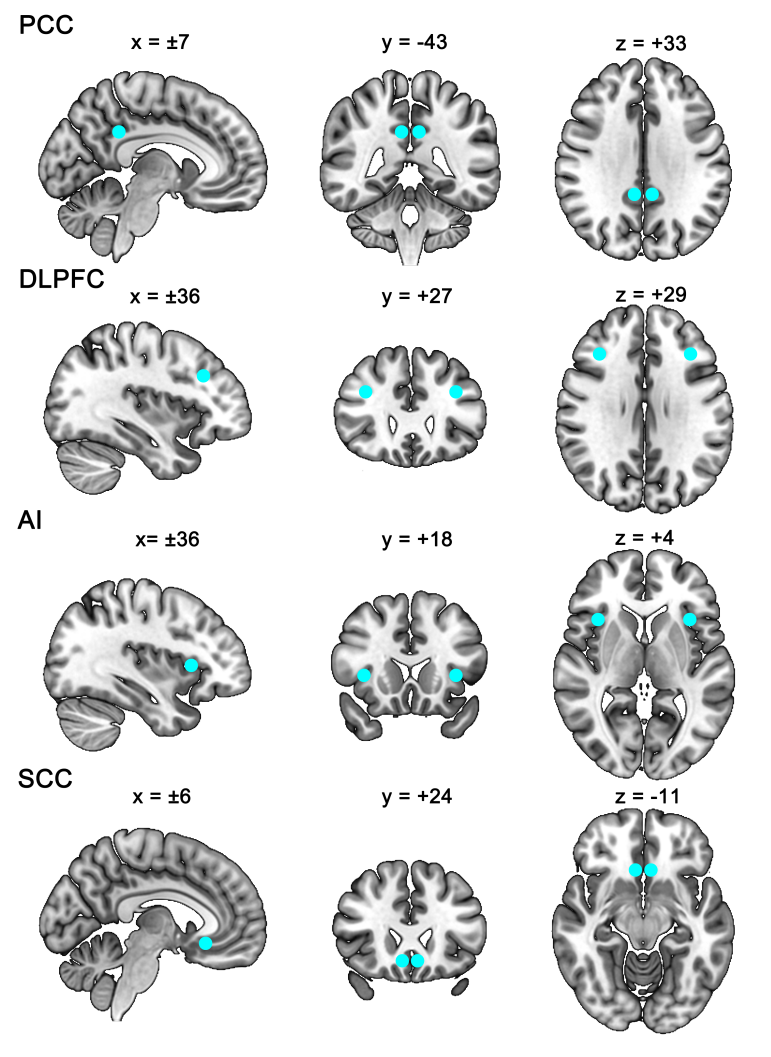
**

**Figure S2.** Eight seeds for the four networks in the bilateral hemisphere. PCC, posterior cingulate cortex; DLPFC, dorsolateral prefrontal cortex; AI, anterior insula; SCC, subcallosal cingulate cortex.

**
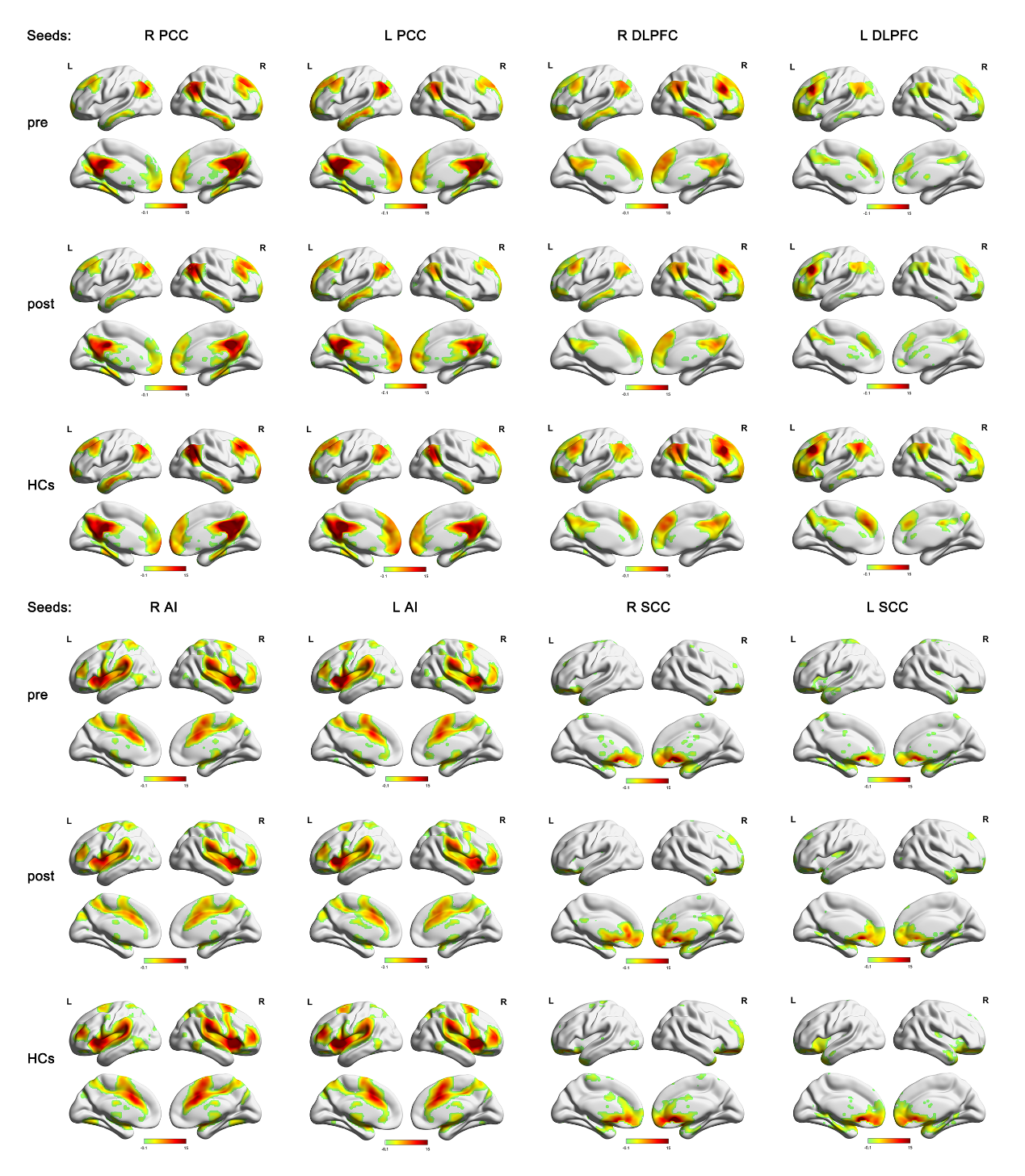
**

**Figure S3.** one-sample *t*-test within-group FC patterns

**
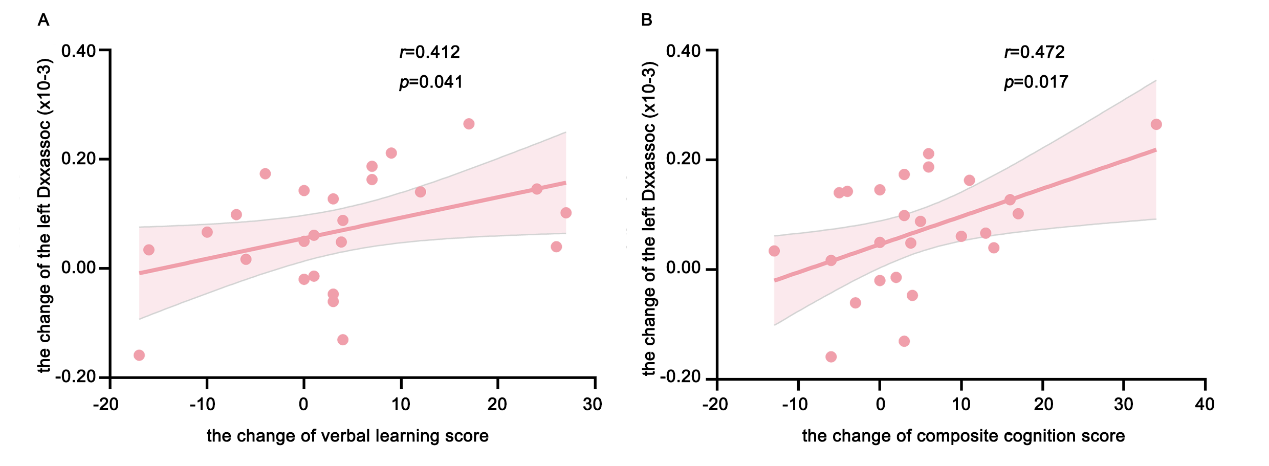
**

**Figure S4.** Correlation analysis for diffusivity values in the x, y, and z directions for projection fibers and association fibers in MDD. MDD, major depressive disorder; D_xxassoc_, diffusivity along the x-axis in the association fibers area.

**Table S1. Demographic for participants in MRI analysis.**

|  | | MDD | | | HCs | *p*-value |
| --- | --- | --- | --- | --- | --- | --- |
| **Demographic** | | | | | | |
| Number of participants | | 25 | 41 | | N/A | |
| Sex (male/female) | | 5/20 | 16/25 | | 0.107† | |
| Age (years) | | 25.76 (6.57) | 23.10 (3.32) | | 0.069 ^a^ | |
| Education (years) | | 15.56 (2.95) | 16.05 (2.51) | | 0.475 ^a^ | |

Mean (S.D.) are reported. MDD, major depressive disorder; HCs, healthy controls; †, The *p* value for gender distribution was obtained by chi-square test; ^a^, the *p* values were obtained by independent-sample *t*-tests; ^*^, *p* < 0.05.

**Table S2. Interobserver agreement on diffusivities and DTI-ALPS** **indexes.**

| Value | Reader 1 | Reader 2 | ICC | 95% CI |
| --- | --- | --- | --- | --- |
| **Left Hemisphere** |  |  |  |  |
| **Association fiber** |  |  |  |  |
| Dxx (×10^−3^ mm^2^/s) | 0.76(0.09) | 0.76(0.10) | 0.895 | 0.846-0.929 |
| Dzz (×10^−3^ mm^2^/s) | 0.37(0.05) | 0.37(0.06) | 0.919 | 0.881-0.946 |
| **Projection fiber** |  |  |  |  |
| Dxx (×10^−3^ mm^2^/s) | 0.67(0.06) | 0.67(0.07) | 0.897 | 0.850-0.930 |
| Dyy (×10^−3^ mm^2^/s) | 0.45(0.05) | 0.44(0.06) | 0.773 | 0.677-0.843 |
| **Right Hemisphere** |  |  |  |  |
| **Association fiber** |  |  |  |  |
| Dxx (×10^−3^ mm^2^/s) | 0.76(0.10) | 0.75(0.10) | 0.903 | 0.857-0.934 |
| Dzz (×10^−3^ mm^2^/s) | 0.37(0.06) | 0.37(0.06) | 0.915 | 0.875-0.942 |
| **Projection fiber** |  |  |  |  |
| Dxx (×10^−3^ mm^2^/s) | 0.68(0.06) | 0.67(0.07) | 0.802 | 0.717-0.864 |
| Dyy (×10^−3^ mm^2^/s) | 0.46(0.05) | 0.46(0.05) | 0.909 | 0.867-0.939 |
| **Left DTI-ALPS index** | 1.75(0.20) | 1.76(0.21) | 0.832 | 0.758-0.885 |
| **Right DTI-ALPS index** | 1.74(0.20) | 1.73(0.24) | 0.844 | 0.774-0.893 |
| **Mean DTI-ALPS index** | 1.75(0.17) | 1.75(0.19) | 0.881 | 0.827-0.919 |

Mean (with standard deviation in parentheses) are reported unless otherwise noted. DTI-ALPS, diffusion tensor image analysis along the perivascular space; ICC, intra-class correlation coefficient.

**Table S3. The significant FC difference in group comparisons (voxel p < 0.005, cluster p < 0.00625, GRF corrected).**

| **seeds** | **voxel *p* < 0.005, cluster *p* < 0.00625, GRF corrected** | | | | | |  |
| --- | --- | --- | --- | --- | --- | --- | --- |
|  | **Significant regions** | **BA** | **voxels** | **MNI** | | | **t** |
|  |  |  |  | **x** | **y** | **z** |  |
| pre vs. HCs | | | | | | | |
| L PCC | R Precuneus | 18 | 67 | 9 | -72 | 24 | 4.3233^a^ |
| pre vs. post | | | | | | | |
| R PCC | L Precuneus | 23 | 130 | -8 | -51 | 24 | -4.54^b^ |
| post vs. HCs | | | | | | | |
| R PCC | L Precuneus | 7 | 454 | -8 | -72 | 48 | -3.60^a^ |

HCs, healthy controls; FC, functional connectivity; GRF, Gaussian random field; BA, Brodmann Area; MNI, Montreal Neurological coordinate; PCC, posterior cingulate cortex; L / R, left / right hemisphere; ^a^, independent-sample t-tests; ^b^, paired-sample *t*-tests.

**Table S4. Differences of** **D_xx_ index in the corpus callosum.**

|  | Pre-treatment MDD (n = 25) | Post-treatment MDD (n = 25) | HCs (n =41) | *p* |
| --- | --- | --- | --- | --- |
| D_xx_ (×10^−2^ mm^2^/s) | 0.158 (0.012) | 0.159 (0.011) | 0.160 (0.010) | > 0.05 ^a, b, c^ |

Mean (S.D.) are reported. MDD, major depressive disorder; HCs, healthy controls; D_xx_, diffusivity along the x-axis. ^a^, difference between pre-treatment MDD and HCs by an independent-sample *t*-tests; ^b^, difference between post-treatment MDD and HCs by an independent-sample *t*-test; ^c^, difference between pre-treatment MDD and HCs by a paired *t*-test.

**Table S5.** **Differences of** **DTI-ALPS indexes between MDD and HCs with D_xx_ index in the corpus callosum included as a nuisance covariate.**

|  | | MDD (n = 25) | HCs (n =41) | *p* | FDR-*p* |  |
| --- | --- | --- | --- | --- | --- | --- |
| **Baseline** | | | | | |  |
| Left DTI-ALPS index | 1.67 (0.13) | 1.78 (0.22) | 0.004^*^ | 0.012^*^ | |  |
| Right DTI-ALPS index | 1.65 (0.13) | 1.73 (0.18) | 0.028^*^ | 0.056 | |  |
| Mean DTI-ALPS index | 1.66 (0.09) | 1.76 (0.18) | 0.002^*^ | 0.012^*^ | |  |
| **Eight-week follow-up** | | | | | | |
| Left DTI-ALPS index | 1.81 (0.25) | 1.78 (0.22) | 0.633 | 0.633 | |  |
| Right DTI-ALPS index | 1.83 (0.28) | 1.73 (0.18) | 0.096 | 0.144 | |  |
| Mean DTI-ALPS index | 1.82 (0.24) | 1.76 (0.18) | 0.224 | 0.269 | |  |

Mean (S.D.) are reported. DTI-ALPS, diffusion tensor imaging along the perivascular space; MDD, major depressive disorder; HCs, healthy controls; D_xx_, diffusivity along the x-axis; FDR, false discovery rate.

**Table S6. Changes of DTI-ALPS indexes after treatment with vortioxetine in MDD** **with D_xx_ index in the corpus callosum included as a nuisance covariate.**

|  | Pre-treatment | Post-treatment | *p* | FDR-*p* |  |
| --- | --- | --- | --- | --- | --- |
| Left DTI-ALPS index | 1.67 (0.13) | 1.81 (0.25) | 0.006^*^ | 0.006^*^ | |
| Right DTI-ALPS index | 1.65 (0.13) | 1.83 (0.28) | 0.003^*^ | 0.005^*^ | |
| Mean DTI-ALPS index | 1.66 (0.09) | 1.82 (0.24) | 0.001^*^ | 0.003^*^ | |

Mean (S.D.) are reported. MDD, major depressive disorder; FDR, false discovery rate; DTI-ALPS, diffusion tensor imaging along the perivascular space; D_xx_, diffusivity along the x-axis. ^*^, *p* < 0.05.

1. August SM, Kiwanuka JN, McMahon RP, Gold JM. The MATRICS Consensus Cognitive Battery (MCCB): clinical and cognitive correlates. *Schizophrenia research.* 2012;134(1):76-82. <https://doi.org/10.1016/j.schres.2011.10.015>.

2. Tombaugh TN. Trail Making Test A and B: normative data stratified by age and education. *Arch Clin Neuropsychol.* 2004;19(2):203-214. <https://doi.org/10.1016/S0887-6177(03)00039-8>.

3. Taoka T, Masutani Y, Kawai H, et al. Evaluation of glymphatic system activity with the diffusion MR technique: diffusion tensor image analysis along the perivascular space (DTI-ALPS) in Alzheimer's disease cases. *Japanese Journal of Radiology.* 2017;35(4):172-178. <https://doi.org/10.1007/s11604-017-0617-z>.

4. Carotenuto A, Cacciaguerra L, Pagani E, Preziosa P, Filippi M, Rocca MA. Glymphatic system impairment in multiple sclerosis: relation with brain damage and disability. *Brain.* 2022;145(8):2785-2795. <https://doi.org/10.1093/brain/awab454>.

5. Lin LP, Su S, Hou W, et al. Glymphatic system dysfunction in pediatric acute lymphoblastic leukemia without clinically diagnosed central nervous system infiltration: a novel DTI-ALPS method. *Eur Radiol.* 2023;33(5):3726-3734. <https://doi.org/10.1007/s00330-023-09473-8>.

6. Frangi AF, Niessen WJ, Vincken KL, Viergever MA. Multiscale vessel enhancement filtering. Paper presented at: Medical Image Computing and Computer-Assisted Intervention — MICCAI’98; 1998//, 1998; Berlin, Heidelberg.

7. Sepehrband F, Barisano G, Sheikh-Bahaei N, et al. Image processing approaches to enhance perivascular space visibility and quantification using MRI. *Sci Rep.* 2019;9(1):12351. <https://doi.org/10.1038/s41598-019-48910-x>.

8. Chan ST, Mercaldo ND, Ravina B, Hersch SM, Rosas HD. Association of Dilated Perivascular Spaces and Disease Severity in Patients With Huntington Disease. *Neurology.* 2021;96(6):e890-e894. <https://doi.org/10.1212/WNL.0000000000011121>.

9. Jenkinson M, Bannister P, Brady M, Smith S. Improved optimization for the robust and accurate linear registration and motion correction of brain images. *NeuroImage.* 2002;17(2):825-841. <https://doi.org/10.1016/s1053-8119(02)91132-8>.

10. Friston KJ, Williams S, Howard R, Frackowiak RS, Turner R. Movement-related effects in fMRI time-series. *Magnetic resonance in medicine.* 1996;35(3):346-355. <https://doi.org/10.1002/mrm.1910350312>.

11. Power JD, Mitra A, Laumann TO, Snyder AZ, Schlaggar BL, Petersen SE. Methods to detect, characterize, and remove motion artifact in resting state fMRI. *Neuroimage.* 2014;84:320-341. <https://doi.org/10.1016/j.neuroimage.2013.08.048>.
